# Mapping the evidence on ultrasonography for temporomandibular joint evaluation in rheumatoid arthritis: a scoping review

**DOI:** 10.1007/s11282-026-00918-6

**Published:** 2026-03-31

**Authors:** Beatriz Braga Tenório, Thaiza Goncalves Rocha, Bruno Augusto Benevenuto de Andrade, Luciana Munhoz, Maria Augusta Visconti, Jefferson R. Tenório

**Affiliations:** 1 Department of Pathology and Oral Diagnosis, School of Dentistry, Universidade Federal do Rio de Janeiro, Rio de Janeiro, RJ Brazil 550 Pedro Calmon Avenue, 21941-630; 2 Department of Medical Imaging, Hematology and Oncology, Medicine School of Ribeirão Preto, University of São Paulo, 3900 Bandeirantes Avenue, Ribeirão Preto, SP 14049-900 Brazil

**Keywords:** Temporomandibular joint disorders, Ultrasonography, Doppler, Ultrasound imaging, Arthritis, Rheumatoid

## Abstract

**Objectives:**

To perform a scoping review on the application of ultrasound (US) for identifying temporomandibular joint (TMJ) alterations in patients with rheumatoid arthritis (RA).

**Methods:**

An electronic search was conducted in PubMed/MEDLINE, Scopus, Web of Science, Embase, and gray literature sources until October 2025, with no language or publication date restrictions. Observational studies and clinical trials evaluating TMJ alterations using US in RA patients were included. Data were collected on study design, sample characteristics, RA-related clinical parameters, TMJ clinical findings, US techniques, and main imaging results.

**Results:**

Initially, a total of 88 records were retrieved. After applying theeligibility criteria, five studies met the inclusion criteria. One hundred and fifty-four patients with RA were evaluated. Most participants were women and had moderate-to-high disease activity. Among the studies reporting age specifically for the RA subgroup, mean ages ranged from 45.4 to 53 years. The US consistently identified joint effusion, synovial thickening, and disc alterations. Only one diagnostic accuracy study reported that US demonstrated 92.4% sensitivity and 90.3% specificity for detecting joint effusion compared with magnetic resonance imaging (MRI). Associations between US findings and clinical indices (DAS28, HAQ) and pain were observed, although inconsistently. Methodological heterogeneity, small sample sizes, and operator dependence were the main limitations.

**Conclusions:**

US is a practical, non-invasive, and affordable adjunctive initial imaging examination for detecting inflammatory TMJ changes in RA. However, it should complement rather than replace MRI, particularly for structural assessment. Future research should prioritize standardized protocols, larger longitudinal cohorts, and integration with clinical and serological data.

## Introduction

Rheumatoid arthritis (RA) is a chronic systemic autoimmune disease defined by persistent synovial inflammation and progressive joint damage, potentially accompanied by extra-articular manifestations [1, 2]. Its etiology involves genetic susceptibility and environmental triggers such as smoking, obesity, and occupational exposures, which contribute to autoantibody production, including rheumatoid factor (RF) and anti-citrullinated protein antibodies (ACPA) [2, 3]. 

RA affects approximately 0.5–1% of the global population, predominantly women, with a peak incidence between 30 and 50 years of age [1, 2]. Clinically, RA usually presents as a symmetric polyarthritis of gradual onset, primarily involving small joints, with pain, swelling, and prolonged morning stiffness [4]. General symptoms such as asthenia, malaise, and low-grade fever are common. In more severe cases, extra-articular involvement may occur, including rheumatoid nodules, interstitial lung disease, and vasculitis [3, 4]. 

The temporomandibular joints (TMJs) may also be affected, with reported prevalence ranging from 40% to 55%, depending on the diagnostic approach and disease duration [5, 6]. TMJ involvement is often underrecognized, partly because of the limited correlation between clinical signs and radiological findings, indicating the need for more reliable diagnostic strategies [5, 6]. Clinically, patients may present with restricted mandibular movement, joint sounds, tenderness, and functional impairment, negatively impacting oral health-related quality of life [5, 7]. 

Magnetic resonance imaging (MRI) remains the reference standard for detecting early inflammatory and soft tissue changes in the TMJ, although its cost and limited accessibility may restrict routine use [6]. Other imaging modalities, such as cone-beam computed tomography (CBCT), are more suitable to bone assessment but are not sensitive for early inflammatory changes [6]. 

Several diagnostic strategies have been investigated to improve the early detection of TMJ changes and facilitate monitoring of TMJ involvement in RA, with a focus on non-invasive and cost-effective methods. Salivary assessment of inflammatory cytokines was initially considered a useful screening tool, but its weak correlation with systemic inflammatory activity limits its clinical value [8]. However, ultrasound (US) has demonstrated favorable cost-effectiveness. Although not sufficient alone as a diagnostic method, US can serve as a helpful adjunct when integrated into a multimodal TMJ assessment in RA [9]. 

A recent systematic review reported that US has acceptable accuracy for detecting the main alterations, including joint effusion and disc displacement [10]. Nevertheless, the authors observed that its performance in evaluating condylar bone changes remains limited and requires further validation using CBCT as the reference standard [10]. Another study reported a positive predictive value of 90% for identifying normal conditions, indicating that US may be useful as a reliable screening tool [9]. US represents a promising ionizing radiation-free adjunct for the initial evaluation of patients with RA.

Despite the growing interest in musculoskeletal US in rheumatology, its application to the evaluation of the TMJ in RA remains limited, inconsistently reported, and methodologically unstandardized. Available studies show wide variation in imaging protocols, operator expertise, diagnostic criteria, and the specific TMJ parameters assessed, which complicates efforts to define the true diagnostic contribution of US in this context. To date, no detailed synthesis has addressed whether TMJ alterations in RA patients can be reliably identified by US. This literature gap reinforces the need to organize and consolidate current knowledge to guide future research. Thus, the objective of this scoping review is to map the existing evidence regarding the use of US in evaluating TMJ involvement in RA and to address the following question: Which alterations can be identified in the TMJs of individuals with RA using US imaging?

## Materials and methods

### Protocol and registration

This scoping review was performed in accordance with the Preferred Reporting Items for Systematic Reviews and Meta-Analyses Extension for Scoping Reviews (PRISMA-ScR) guidelines (https://www.equator-network.org/reporting-guidelines/prisma-scr/). The review protocol was registered on the Open Science Framework (10.17605/OSF.IO/UQWSH).

### Search strategies

For this scoping review, an extensive literature search was conducted using the COCOPOP (Condition–Context–Population) strategy. In October 2025, systematic searches were performed in electronic databases, including PubMed/MEDLINE, Scopus, Web of Science, and Embase, without any restrictions on language or publication date. Additional sources were explored through gray literature platforms (OpenGrey and Google Scholar), as well as in the reference lists of the selected studies. For the gray literature, OpenGrey and Google Scholar were searched using the terms “Temporomandibular Joint Disorders” AND “Ultrasonography” AND “Arthritis, Rheumatoid”. In Google Scholar and OpenGrey, the first 200 results sorted by relevance were screened.

After completion of the initial search, automatic monthly alerts were activated in each database using the native “save search” and email notification functions (e.g., the “Create alert” feature in PubMed via an NCBI account), allowing the identification of newly indexed studies. The full electronic search strategies for all databases, including controlled vocabulary terms (MeSH and Emtree) and free-text keywords adapted to the syntax of each platform, are detailed in Table 1.

**Table 1:** Search strategies

Database	Search
PubMed/MEDLINE	(“Temporomandibular Joint Disorders“[MeSH Terms] OR “Temporomandibular Joint Disorders“[Title/Abstract] OR “Temporomandibular Disorder“[Title/Abstract] OR “Temporomandibular Disorders“[Title/Abstract] OR “Temporomandibular Joint Disorder“[Title/Abstract] OR “Temporomandibular Joint Dysfunctions“[Title/Abstract] OR “TMJ Disorders“[Title/Abstract]) **AND** (“Ultrasonography“[MeSH Terms] OR “Endosonography“[MeSH Terms] OR “Ultrasonography, Doppler“[MeSH Terms] OR “Ultrasonography“[Title/Abstract] OR “Endosonography“[Title/Abstract] OR “Ultrasonography, Doppler“[Title/Abstract] OR “Echographies“[Title/Abstract] OR “Echography“[Title/Abstract] OR “Sonogram“[Title/Abstract] OR “Sonograms“[Title/Abstract] OR “Sonographic Imaging“[Title/Abstract] OR “Sonographies“[Title/Abstract] OR “Sonography“[Title/Abstract] OR “Ultrasonic Imaging“[Title/Abstract] OR “Ultrasonographies“[Title/Abstract] OR “Ultrasound Imaging“[Title/Abstract]) **AND** (“Arthritis, Rheumatoid“[MeSH Terms] OR “Caplan Syndrome“[MeSH Terms] OR “Felty Syndrome“[MeSH Terms] OR “Rheumatoid Nodule“[MeSH Terms] OR “Rheumatoid Vasculitis“[MeSH Terms] OR Felty Syndrome[Title/Abstract] OR “Felty’s Syndrome“[Title/Abstract] OR “Rheumatoid Arthritis“[Title/Abstract] OR “Rheumatoid Nodule“[Title/Abstract] OR “Rheumatoid Nodules“[Title/Abstract])
Web of Science	TS=(“Temporomandibular Joint Disorders” OR “Temporomandibular Disorder” OR “Temporomandibular Disorders” OR “Temporomandibular Joint Disorder” OR “Temporomandibular Joint Dysfunctions” OR “TMJ Disorders”) **AND** TS=(“Ultrasonography” OR “Endosonography” OR “Ultrasonography, Doppler” OR “ethographies” OR “Echography” OR “Sonogram” OR “Sonograms” OR “Sonographic Imaging” OR “sinographies” OR “Sonography” OR “Ultrasonic Imaging” OR “Ultrasonographies” OR “Ultrasound Imaging”) **AND** TS=(“Arthritis, Rheumatoid” OR “Caplan Syndrome” OR “Felty Syndrome” OR “Felty’s Syndrome” OR “Rheumatoid Arthritis” OR “Rheumatoid Nodule” OR “Rheumatoid Nodules” OR “Rheumatoid Vasculitis”)
Scopus	(INDEXTERMS (“Temporomandibular Joint Disorders” OR “Temporomandibular Disorder” OR “Temporomandibular Disorders” OR “Temporomandibular Joint Disorder” OR “Temporomandibular Joint Dysfunctions” OR “TMJ Disorders”) OR TITLE-ABS-KEY (“Temporomandibular Joint Disorders” OR “Temporomandibular Disorder” OR “Temporomandibular Disorders” OR “Temporomandibular Joint Disorder” OR “Temporomandibular Joint Dysfunctions” OR “TMJ Disorders”)) **AND** (INDEXTERMS (“Ultrasonography” OR “Endosonography” OR “Ultrasonography, Doppler” OR “Echographies” OR “Echography” OR “Sonogram” OR “Sonograms” OR “Sonographic Imaging” OR “Sonographies” OR “Sonography” OR “Ultrasonic Imaging” OR “Ultrasonographies” OR “Ultrasound Imaging”) OR TITLE-ABS-KEY (“Ultrasonography” OR “Endosonography” OR “Ultrasonography Doppler” OR “Echographies” OR “Echography” OR “Sonogram” OR “Sonograms” OR “Sonographic Imaging” OR “Sonographies” OR “Sonography” OR “Ultrasonic Imaging” OR “Ultrasonographies” OR “Ultrasound Imaging”)) **AND** (INDEXTERMS (“Arthritis, Rheumatoid” OR “Caplan Syndrome” OR “Felty Syndrome” OR “Rheumatoid Nodule” OR “Rheumatoid Vasculitis”) OR TITLE-ABS-KEY (“Arthritis, Rheumatoid” OR “Caplan Syndrome” OR “Felty Syndrome” OR “Felty&apos;s Syndrome” OR “Rheumatoid Arthritis” OR “Rheumatoid Nodule” OR “Rheumatoid Nodules” OR “Rheumatoid Vasculitis”))
EMBASE	(‘temporomandibular joint disorders’/exp OR ‘temporomandibular joint disorders’ OR ‘temporomandibular joint disorders’:ab, ti OR ‘temporomandibular disorder’:ab, ti OR ‘temporomandibular disorders’:ab, ti OR ‘temporomandibular joint disorder’:ab, ti OR ‘temporomandibular joint dysfunctions’:ab, ti OR ‘tmj disorders’:ab, ti) **AND** (‘ultrasonography’/exp OR ‘ultrasonography’ OR ‘endosonography’/exp OR ‘endosonography’ OR ‘ultrasonography doppler’/exp OR ‘ultrasonography doppler’ OR ‘ultrasonography’:ab, ti OR ‘endosonography’:ab, ti OR ‘ultrasonography doppler’:ab, ti OR ‘echographies’:ab, ti OR ‘echography’:ab, ti OR ‘sonogram’:ab, ti OR ‘sonograms’:ab, ti OR ‘sonographic imaging’:ab, ti OR ‘sonographies’:ab, ti OR ‘sonography’:ab, ti OR ‘ultrasonic imaging’:ab, ti OR ‘ultrasonographies’:ab, ti OR ‘ultrasound imaging’:ab, ti) **AND** (‘arthritis, rheumatoid’/exp OR ‘arthritis, rheumatoid’ OR ‘caplan syndrome’/exp OR ‘caplan syndrome’ OR ‘felty syndrome’/exp OR ‘felty syndrome’ OR ‘rheumatoid nodule’/exp OR ‘rheumatoid nodule’ OR ‘rheumatoid vasculitis’/exp OR ‘rheumatoid vasculitis’ OR ‘arthritis, rheumatoid’:ab, ti OR ‘caplan syndrome’:ab, ti OR ‘felty syndrome’:ab, ti OR ‘rheumatoid arthritis’:ab, ti OR ‘rheumatoid nodule’:ab, ti OR ‘rheumatoid nodules’:ab, ti OR ‘rheumatoid vasculitis’:ab, ti)
Open Grey	“Temporomandibular Joint Disorders” **AND** “Ultrasonography” **AND** “Arthritis, Rheumatoid”
Google Scholar	“Temporomandibular Joint Disorders” **AND** “Ultrasonography” **AND** “Arthritis, Rheumatoid”

### Eligibility criteria

The inclusion criteria were established according to the COCOPOP strategy and defined as follows: Condition (CO): TMJ alterations; Context (CO): assessed using US imaging; Population (POP): individuals diagnosed with RA.

Eligible studies included observational designs (cross-sectional, cohort, and case-control) and clinical trials evaluating TMJ changes using US in patients with RA, regardless of disease activity, age, sex, or ethnicity. Exclusion criteria included case reports, case series, conference abstracts, letters to the editor, narrative or systematic reviews, animal or in vitro studies, and studies involving participants with other concomitant autoimmune or musculoskeletal diseases affecting the TMJ.

### Study selection

All references retrieved from the databases were imported into the EndNote reference manager (X7 online version; Thomson Reuters, Philadelphia, PA) to remove duplicates and subsequently transferred to Rayyan software (https://www.rayyan.ai) for further screening. After another round of the automatic and manual duplicate removal, two reviewers independently evaluated the titles and abstracts based on the eligibility criteria. When the available information was insufficient, the full text was obtained for further assessment. All potentially eligible articles were read in full to confirm inclusion. Discrepancies were resolved through discussion and consensus, with the intervention of a third reviewer when necessary. Articles published in languages other than English, Portuguese, and Spanish were translated using automated tools. For manuscripts without accessible full texts, the corresponding authors were contacted by email up to three times before exclusion.

### Data extraction and synthesis

From each included study, the following information was extracted: author, year of publication, country, study design, sample size, sex, age (mean, median, and range), RA-related clinical data, TMJ clinical data, and main US findings. When available, details regarding the US technique and image acquisition protocols were also collected. When data were incomplete or unclear, the corresponding authors were contacted by email once per week for three consecutive weeks to request further information.

The features of the included studies were summarized in tabular format. For the synthesis, data were grouped and analyzed descriptively.

## Results

**Fig. 1:**
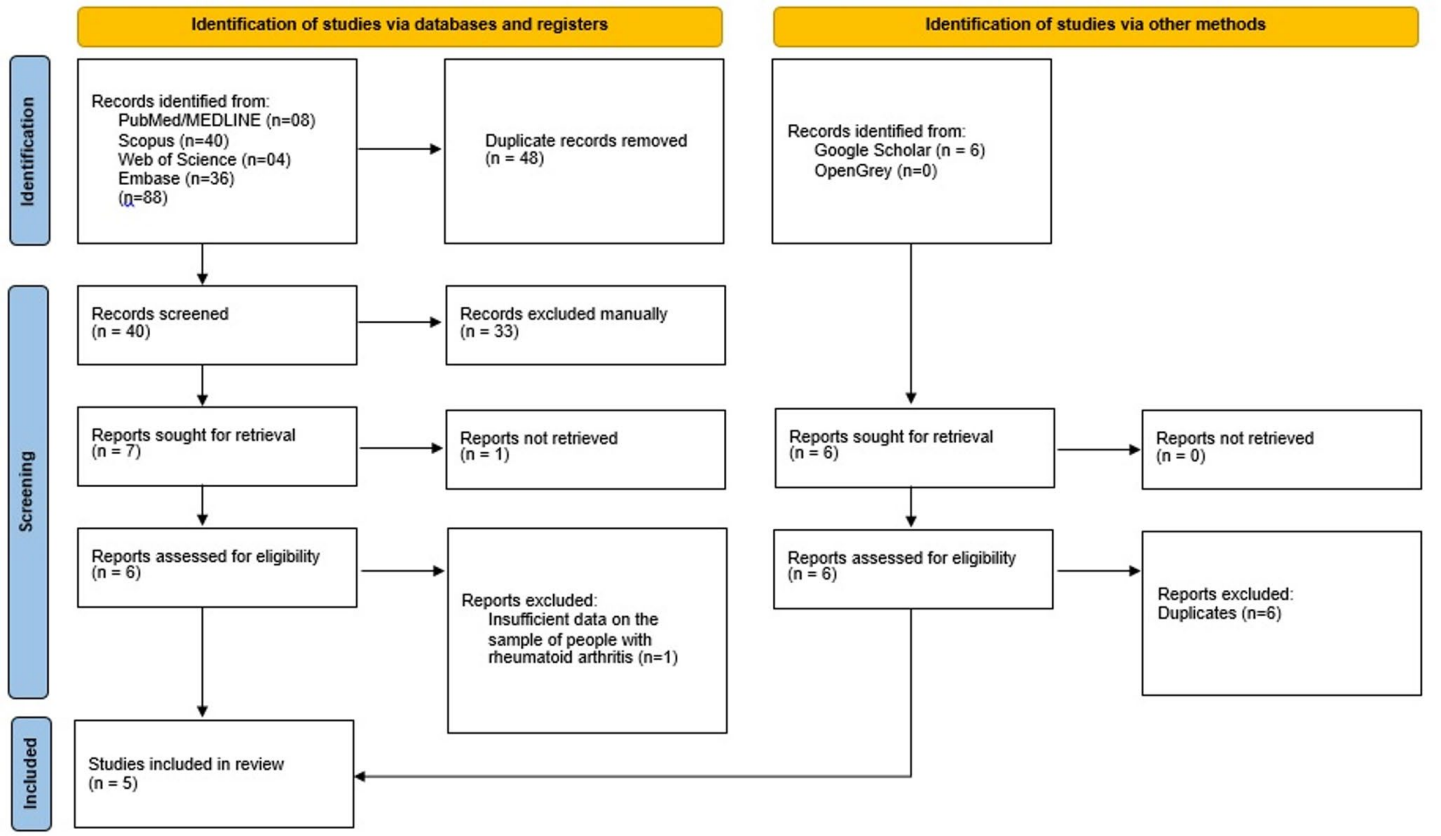
PRISMA flowchart

A total of 88 records were initially retrieved from the primary databases. After automatic and manual removal of duplicates, 40 titles and abstracts were screened. From this group, 33 were excluded for not meeting the eligibility criteria, leaving seven articles selected for full text assessment. One study could not be accessed despite repeated attempts to contact the corresponding author [11], while another [12] did not report absolute data for the RA group. An additional six records were identified through gray literature searches; however, all were duplicates of articles already retrieved from the main databases. As a final result, five studies [13–17]were included. The study selection process is illustrated in Fig. 1. A summary of our findings is available on Table 2.

**Table 2:** Main features of the included studies

Author, Year, Country, Study Design	*N*	Age	Sex	RA related data	ATM clinical data in RA individuals	Main ultrasound data
Melchiorre et al., 2003, Italy, Coss-sectional [13]	RA: 22	RA: UD	RA: UD	All patients had active disease. ESR mean: 43.8 mm/h. C-reactive protein (mean concentration): 7.05 mg/dL	Pain and functional impairment: *n* = 12 (54,54%)	Disc alterations (anteriorly displaced and hypoechoic): *n* = 7 (31,81%) Disc morphological changes were not well defined by ultrasound. Condylar changes: *n* = 16 (72,72%) Effusion: *n* = 12 (54,54%)
EL-Melegy et al., 2017, Egypt, Cross-sectional [14]	RA: 20	RA: mean 47.3 ± 10.03	RA: 2 M, 18 F	DAS28 mean: 5.5 ± 1.4	Pain: *n* = 27 (67.5%)* Tenderness: *n* = 27 (67.5%)* Mouth opening limitation: *n* = 20 (50%)*	Erosion: *n* = 23 (57,5%); Efusion: *n* = 25 (62,5%); Disc displacement: *n* = 21 (52,5%).
Elshoura et al., 2020; Egypt, Non-randomized Clinical Trial [15]	RA: 11	RA: mean 45.4 ± 9.9 (Min: 20; Max: 56)	RA: 11 F	DAS28 mean: 5.6 ± 1.2 (Min: 4; Max:7). Disease duration (years): 9.5 ± 4.1 (Min: 2; Max: 14).	Tenderness: Média: 4 (score 0–4) Crackling: 1.14 ± 0.65 (score 0–2)	12 (57.1%)* TMJs had synovial effusion at baseline (before perineural dextrose injection therapy) and 09 (42.8%)* TMJs had synovial effusion 4 weeks after application (*p* = 0.57).
Becenen Durmuş et al., 2025, Turkey, Cross-sectional [16]	RA: 51 CG: 51	RA: mean 53.0 ± 10.4 (Min: 18; Max: 65) CG: mean 51.3 ± 6.9 (Min: 18; Max: 65)	RA: 16 M, 35 F CG: 16 M, 35 F	DAS28 mean: 3.37 ± 1.25	Pain: *n* = 29 (26,9%) RDD: 6 (11,8%) NRDD: 3 (5,9%) DJF: 5 (9,8%)	The RA group showed greater articular disc thickness than the CG (*p* < 0.001). Effusion in the right and left TMJ was observed in 7.8% of patients with RA, being more frequent than in the CG (*p* = 0.169 and *p* = 0.041).
Guiducci et al., 2025, Italy, prospective diagnostic accuracy [17]	RA: 50	RA: UD	RA: UD	RA: UD	RA: UD	Disc displacement: *n* = 38 (76%) Erosion: *n* = 14 (28%) Efusion: *n* = 44 (88%) Condylar alterations: *n* = 26 (52%)

Legends – RA: Rheumatoid arthritis; CG: Control group; Min: Minimum value; Max: Maximum value; DAS28: Disease activity score-28; TMJ: Temporomandibular joint; TMD: Temporomandibular disorders; RDD: Reduced disc displacement; NRDD: Non-reduced disc displacement; DJD: Degenerative joint disease; UD: Unavailable data; M: male; F: female; ESR: Erythrocyte sedimentation rate. * The author considered each TMJ as a sampling unit in this evaluation

### General characteristics of the studies

Among the included studies, three [13, 14, 16] had a cross-sectional design, one [17] was a prospective diagnostic accuracy study, and one [15] was a non-randomized clinical trial, published between 2003 [13] and 2025 [17]. These investigations were conducted by research groups from Italy [13, 17], Egypt [14, 15] andTurkey [16]. In total, 152 individuals with RA were evaluated. Sample sizes in the cross-sectional studies ranged from 18 to 51 participants, while the clinical trial enrolled 11 participants. A predominance of female participants was reported in three studies [14–16]. Mean age ranged from 45.4 years [15] to 53 years [16] among studies that reported age specifically for the RA subgroup. Two studies [13, 17] reported only overall sample means, without providing age data restricted to the RA group.

### Clinical and laboratorial AR parameters

The included studies diagnosed patients with RA based on the 2010 American College of Rheumatology/European Alliance of Associations for Rheumatology (ACR/EULAR) classification criteria [14–16]. Disease duration varied considerably, with reported means ranging from 9.5 ± 4.1 years [15] to 13.4 ± 8.9 years [16]. Disease activity, measured using the *Disease Activity Score 28* (DAS28), ranged from remission to severe activity: in one study [16], 17.6% of patients were in remission, 25.5% had low activity, 47% had moderate activity, and 9.9% presented with severe disease. Another investigation [14] described a mean DAS28 of 5.5 ± 1.4, indicating high activity, and Elshoura et al. (2020) [15] reported a similarly elevated value (5.6 ± 1.2). Functional status reflected this trend: the mean Health Assessment Questionnaire (HAQ) scores was 0.59 in the Turkish study [16], whereas higher modified HAQ scores of 1.1 and 1.5 were mentioned in the Egyptian cohorts [14, 15]. Seropositivity was also frequent, with more than 80% of patients testing positive for RF and anti-CCP antibodies [16]. Inflammatory markers, such as erythrocyte sedimentation rate (ESR) and C-reactive protein (CRP), were frequently elevated, particularly in studies that included patients with more active disease [14, 16]. 

Regarding treatment, patients with refractory TMJ involvement were under systemic therapy, usually with methotrexate, leflunomide, hydroxychloroquine, and, in some cases, biologic agents such as the anti-TNF drugs (etanercept), in addition to non-steroidal anti-inflammatory drugs (NSAIDs) for pain relief [15]. Another study mentioned the use of disease-modifying antirheumatic drugs (DMARDs) and corticosteroids as part of the therapeutic strategy [13]. However, neither the Turkish study [16] nor the study by El-Melegy et al. (2017) specified the antirheumatic regimens adopted in their cohorts [14]. 

### TMJ clinical data

Clinical evaluation of TMJ involvement in RA patients included symptom assessment, functional testing, and structured questionnaires. The most commonly reported manifestations were pain, tenderness, joint sounds (clicking or crepitus), and restricted mandibular movements [14, 15]. Several studies applied standardized tools: the *Diagnostic Criteria for Temporomandibular Disorders* (DC/TMD) form, which includes both physical and psychosocial domains [16], and Fonseca’s questionnaire to quantify dysfunction [14, 15]. Pain intensity was usually measured using the visual analogue scale (VAS), and maximum mouth opening was assessed with a ruler, with values under 4 cm considered abnormal [14, 15]. Additional clinical tests included palpation of the masticatory muscles, evaluation of joint locking, the presence of crepitus, and deviations during mandibular movement [15, 16]. Functional limitations related to TMJ symptoms were assessed using the HAQ or mHAQ scales [14–16]. 

### Data related to the US technique

US techniques varied considerably among the included studies, although some methodological similarities were observed. Melchiorre et al. (2003) [13] employed a 7.5 MHz linear probe with a 1 cm spacer, conducting both static and dynamic bilateral assessments bilaterally. The examiner was blinded to the MRI results, and intra-observer reliability was assessed. Guiducci et al. (2025) [17] used a high-frequency linear probe (12–16 MHz, MyLABX8 eXP, Esaote) following a structured “working table” protocol that standardized probe positioning in both longitudinal and coronal planes during static and dynamic phases. All examinations were bilateral, performed by a single experienced professional, and results were compared with MRI as the reference standard [17]. 

In the Egyptian investigations, El-Melegy et al. (2017) [14] performed bilateral scans in both closed- and open-mouth positions using a 7.5–12 MHz linear probe (optimal at 9 MHz). Patients were in the supine position, and images were acquired by a radiologist and a rheumatologist, both trained in musculoskeletal US imaging. The evaluation protocol included transverse and longitudinal sections with semi-quantitative assessment of joint effusion, measurement-based evaluation of erosions, and indirect analysis of disc position using the capsule–condyle distance. Similarly, Elshoura et al. (2020) [15] employed a 7–12 MHz linear probe (optimal at 12 MHz, LOGIQ P5 R4.0, GE), applying both grey-scale and power Doppler technique. Bilateral examinations were also performed with the patient in supine position and conducted by an experienced rheumatologist. Standardized scoring systems from OMERACT were adopted to evaluate effusion, synovial thickening, erosions, and disc position.

Becenen Durmuş et al. (2025) [16] adopted a distinct protocol using a LOGIQ 9 system (GE Healthcare) with a 7–12 MHz linear transducer. The examination applied static imaging, limited to the closed-mouth position. The TMJs were scanned bilaterally, with specific measurements of disc and masseter muscle thickness at rest. All scans were performed by a single experienced sonographer who was blinded to the clinical data [16]. 

Taken together, these technical variations reflect the absence of a standardized TMJ-specific US protocol. Differences in transducer frequency, probe positioning, dynamic assessment, and diagnostic criteria may have influenced the variability in reported findings and diagnostic performance among studies. This heterogeneity confirms the operator-dependent nature of the method and the need for standardized acquisition and interpretation parameters to improve reproducibility in future research.

### Data and clinical correlations

US proved to be a useful tool for detecting TMJ involvement in patients with RA, consistently identifying joint effusion, synovial thickening, erosions, and disc abnormalities. In the Turkish study [16], US revealed increased disc thickness in patients with TMJ pain, and a diagnostic cut-off value of 1.55 mm was proposed to indicate dysfunction. In Egyptian investigations, musculoskeletal US detected TMJ erosions in 57.5% of cases, effusion in 62.5%, and disc displacement in 52.5%, with diagnostic performance comparable to MRI, although MRI still showed slightly higher detection rates (82.5% vs. 77.5%) [14]. In the non-randomized clinical trial, patients receiving perineural injection therapy showed symptomatic improvement; however, US continued to reveal persistent structural abnormalities, suggesting limitations in assessing tissue repair [15]. An earlier study by Melchiorre et al. (2003) [13] found that US could detect pathological changes in a larger number of cases than MRI, although its specificity (75.0%) was lower, particularly for condylar changes. More recently, Guiducci et al. (2025) demonstrated high sensitivity (92.4%) and specificity (90.3%) of US for joint effusion compared with MRI, while performance was reduced for disc displacement (72%) and condylar surface irregularities (53%), indicating that diagnostic capacity varies depending on the anatomical parameter evaluated [17]. 

Some studies addressed the relationship between US findings and clinical parameters. In the study by El-Melegy et al. (2017) [14], effusion and disc displacement detected both by US and MRI were significantly associated with disease activity scores (DAS28) and functional status (mHAQ), whereas erosions identified by US alone did not consistently correlate with clinical measures. Pain intensity, joint tenderness, and functional limitations also associated with US evidence of TMJ involvement. However, structural changes were identified in patients without clinical symptoms, suggesting that US can detect imaging abnormalities before the onset of symptoms [14, 16]. 

## Discussion

The nature of TMJ disorders is inherently complex, particularly when associated with rheumatic conditions. TMJ involvement in RA can be difficult to diagnose, as inflammatory and degenerative changes frequently occur simultaneously, and clinical findings do not consistently match structural abnormalities. In this situation, accessible and non-invasive diagnostic tools become particularly relevant, especially within limited healthcare systems. US has emerged as a valuable imaging modality in rheumatology, and this review demonstrates that it can be applied to the evaluation of the TMJs in patients with RA. Within rheumatologic practice, high-resolution musculoskeletal US has proven effective in detecting subclinical synovitis and in predicting both disease recurrence and structural progression [18, 19]. Grayscale US allows for the assessment of synovial thickening, while Doppler evaluation identifies inflammatory activity and neoangiogenesis [19]. The integration of US into clinical protocols has improved the sensitivity of the 2010 ACR/EULAR classification criteria, helping to identify patients likely to benefit from early treatment with methotrexate [18]. 

Most of the selected studies included RA patients with moderate to high disease activity, as indicated by elevated DAS28 scores, frequent seropositivity for RF and anti-CCP antibodies, and increased inflammatory markers. This pattern suggests a possible selection bias toward more symptomatic individuals, which may have influenced the high rate of US-detected alterations. However, from a rheumatologic perspective, US is recommended to improve diagnostic accuracy in cases of suspected RA with diagnostic uncertainty [1, 2, 19]. According to EULAR, this recommendation is supported by evidence showing that US is superior to clinical examination in up to 75% of cases [19–21]. In addition, studies in early oligoarthritis have shown that US identifies a greater number of patients who later develop polyarthritis compared with clinical assessment alone, thereby improving RA classification [20]. 

Beyond the US ability to detect joint alterations, there remains a clear need for methodological standardization. Parameters such as transducer frequency, examination position (supine or seated, mouth open or closed, static or dynamic images), operator expertise (oral radiology specialists, rheumatologists, or US professionals), and image acquisition protocols vary widely among studies. This inconsistency, also observed in the investigations included in the present scoping review, may have contributed to differences in the reported outcomes. Considering the aforementioned, a previous publication [20] specifically highlighted the impact of operator experience on US image acquisition and interpretation, emphasizing how variability in training and technical execution may directly influence diagnostic performance. Similarly, technical aspects of the procedure have not been systematically explored within the intersection of rheumatology, radiology, and oral medicine.

In our scoping review, US consistently demonstrated its utility in detecting joint effusion and periarticular inflammatory changes. However, its accuracy was lower for assessing disc position and osseous alterations compared with MRI. Importantly, disc displacement is not the primary pathological feature in RA, which is fundamentally driven by autoimmune-mediated synovial inflammation [1, 18]. Disc displacement is highly prevalent in the general population, is frequently diagnosed based on clinical criteria, and often does not require specific intervention [24]. Therefore, the lower diagnostic performance of US for disc position should not be interpreted as a major limitation in the context of RA.

Although disease mechanisms differ between adult RA and juvenile inflammatory conditions, the performance pattern of US in detecting synovitis-related alterations appear consistent for inflammatory disorders. In pediatric cohorts, Kirkhus et al. demonstrated that US reliably detected synovitis-related changes, such as capsular widening, showing moderate correlation and acceptable agreement with MRI, despite measurement variability near technical thresholds [22]. A meta-analysis further reinforced these findings: Zaman et al.(2024) [23] showed that while US performs well in identifying effusion and active inflammation, MRI remains more effective in the overall detection of TMJ dysfunction, particularly for structural abnormalities. In parallel, Su et al. [24] concluded that although high-resolution US offers modest additional value in confirming or ruling out disc displacement, its diagnostic accuracy remains technique-dependent and limited. Emerging evidence suggests that the combination of effusion, synovial hypertrophy and power Doppler signal may represent a more specific inflammatory ultrasound pattern, whereas structural findings such as cartilage thinning, cortical irregularities and enthesophytes appear in both inflammatory and non-inflammatory conditions.[26] The distinction between inflammatory and mechanical phenotypes therefore relies on pattern recognition rather than on single imaging features.

Imaging is essential for the management of RA, as therapeutic decisions are increasingly guided by objective evidence of inflammatory activity and structural damage [1, 18, 19]. In the early stages, the identification of synovitis may support the prompt initiation or escalation of disease-modifying antirheumatic drugs, whereas the detection of structural deterioration can influence long-term therapeutic strategies [18, 19]. In the context of TMJ, imaging findings may help distinguish predominantly inflammatory conditions, potentially responsive to systemic pharmacologic control, from advanced structural alterations that may require local interventions or, in selected cases, surgical management [6]. Given that clinical manifestations do not consistently correlate with imaging findings in TMJ involvement [5, 6], appropriate imaging assessment becomes particularly relevant for defining the most suitable treatment approach and for monitoring response over time.

US presents practical advantages that justify its inclusion in the diagnostic pathway of TMJ involvement in RA. It is a non-invasive, radiation-free, relatively low-cost modality that allows dynamic, real-time evaluation, with greater accessibility compared with MRI, particularly in public healthcare settings [6, 9, 10]. Moreover, its ability to detect joint effusion and synovial changes may support the early identification of inflammatory activity. However, important limitations must be acknowledged. US is highly operator-dependent, lacks standardized acquisition protocols, and demonstrates reduced accuracy for assessing disc position and osseous alterations when compared with MRI [10, 23, 24]. The challenges related to operator dependence are conrcted with the absence of standardized acquisition and measurement protocols. Insights from the pediatric literature on TMJ US in juvenile idiopathic arthritis further illustrate how variations in scan plane, measurement level (condylar versus subcondylar), and the use of non-validated cut-off values for capsular width can substantially affect diagnostic performance and correlation with MRI [22]. Additionally, methodological variability among studies may further limit reproducibility. For this reason, US should be regarded as a complementary modality rather than a substitute for comprehensive imaging evaluation.

The evidence synthesized in this scoping review should be interpreted with caution. Most studies adopted cross-sectional designs and included relatively small samples, limiting both the robustness and applicability of their conclusions. Moreover, important clinical factors, such as the influence of long-term pharmacological treatments and the presence of comorbidities, were not consistently explored.

Although some studies reported associations between joint effusion or disc displacement and clinical measures (DAS28, mHAQ, pain, functional limitation), the overall clinical utility of US in the assessment of TMJ involvement in RA remains insufficiently defined. The variability in reported associations between US findings and systemic clinical indices may reflect the fact that TMJ involvement does not necessarily parallel overall disease activity. Importantly, DAS28 does not include TMJ assessment, and localized joint inflammation may occur independently of composite systemic scores. These factors limit the ability to draw firm conclusions regarding clinical-US correlations.

It is important to emphasize that degenerative TMJ changes may also be observed in non-rheumatologic conditions, including TMJ disorders in the general population. As only one included study incorporated a healthy control group, and few studies performed direct comparisons with MRI, the current evidence remains insufficient to determine the discriminative capacity of US in differentiating inflammatory RA-related alterations from degenerative changes. Future controlled diagnostic accuracy studies are necessary to address this distinction more definitively.

As this study was designed as a scoping review, no formal risk of bias assessment was conducted; however, key methodological characteristics of the included studies were considered during the interpretative synthesis to contextualize the findings. The lack of clearly defined inclusion criteria and insufficient detail regarding the sampling strategy raise concerns about selection bias and representativeness. In many studies, exposure and outcome measures, particularly clinical assessments and image-based TMJ evaluations, were not sufficiently standardized or lacked evidence of validity and reliability, which may compromise measurement accuracy. Additionally, examiners were often not blinded to the participants’ clinical status, increasing the potential for observer bias. Important confounding factors, such as disease duration, inflammatory activity, pharmacological treatments, and dental or occlusal conditions, were not uniformly identified or controlled in the analyses. Finally, incomplete description of participant features, insufficient details on data management, and lack of transparency in statistical methods further restricted the interpretation and comparison of results. In combination, these issues point to the need for more rigorous methodological design and reporting standards in future research.

## Conclusion

US is a promising adjunct imaging examination in the evaluation of TMJ involvement in RA, particularly for identifying joint effusion, synovial thickening, and early inflammatory changes. However, the limited sample sizes and methodological heterogeneity of the included studies reduce the strength and generalization of the current evidence. Although US offers advantages such as lower cost and the absence of ionizing radiation, its diagnostic performance remains inferior to MRI for evaluating disc position and bone alterations. Future research should prioritize well-designed longitudinal studies, standardized imaging protocols, and integration with clinical and serological measures to better define the diagnostic and prognostic value of US in this scenario.

## Data Availability

The data extracted and analyzed during this scoping review are available from the corresponding author upon reasonable request.
